# Central obesity and other factors associated with uncontrolled asthma in women

**DOI:** 10.1186/s13223-015-0076-y

**Published:** 2015-03-31

**Authors:** Albertina Varandas Capelo, Vania Matos de Fonseca, Maria Virgínia Marques Peixoto, Sonia Regina de Carvalho, Larissa Garcia Guerino

**Affiliations:** Gaffrée and Guinle University Hospital of Federal University of the State of Rio de Janeiro, Rio de Janeiro, Brazil; Institute of women, children and adolescents Health Fernandes Figueira – IFF-FIOCRUZ, Rio de Janeiro, Brazil

**Keywords:** Asthma control, Women, Central obesity, Comorbidities

## Abstract

**Background:**

Asthma remains an uncontrolled disease. The Comorbidities, particularly obesity, and several other factors have been identified as being individually associated with asthma control, and these factors vary among different countries and between sexes. Studies have suggested that the harmful effects of these factors are greatest among women. The aim of the present study was to identify associated factors with uncontrolled asthma in women at the outpatient clinic of a Federal University Hospital in Rio de Janeiro, Brazil.

**Methods:**

Cross-sectional study with asthmatic women, older than 18 years old. All subjects were included according to stringent criteria. The study used a structured questionnaire with data about demographic variables, education level, comorbid conditions, and disease history. Anthropometric and spirometric measurements were obtained. Asthma control was assessed by three different tools: the Asthma Control Test (ACT), the Asthma Control Questionnaire (ACQ) and the Global Initiative for Asthma (GINA) criteria.

**Results:**

A total of 124 women were included, and 57%, 38% and 21% were totally controlled according to ACT, ACQ and GINA criteria, respectively. A total of 31.5% were obese. According to the CI (Conicity Index) and WC (waist circumference) respectively, 84% and 68% were centrally obese. There was no association between asthma control and age, education, duration of the disease or BMI (Body Mass Index) in the three models, but there was a significant association between central obesity and asthma control with the ACQ and GINA assessment tools, controlling for explanatory variables such as GERD (gastroesophageal reflux disease). Pre-bronchodilator percent predicted forced expiratory volume in one second (FEV_1_) and forced vital capacity (FVC) were significantly associated with age and FVC was also associated with central obesity.

**Conclusions:**

Asthma remains uncontrolled in women despite treatment, and central obesity seems to have a negative influence on the control of the disease. We believe that women should be studied as a separate group and suggest prospective studies with assessment of fat distribution and biomarkers, controlling for possible comorbidities associated with asthma control.

## Introduction

Asthma is a chronic respiratory disease that remains an important cause of morbidity and mortality worldwide [1,2]. Although some studies suggest that the prevalence of asthma might be stabilising or even decreasing in Western countries, it has been described as increasing in prevalence in Latin America, Africa and Asia [3]. Brazil has the sixth highest asthma prevalence, with 20 million asthmatics, responsible for a fourth of all hospitalisations according to the Brazilian Health System database (DATASUL) [4]. Asthma is an inflammatory and complex disease with many aetiologic mechanisms and, a natural history that is poorly understood. Despite this, there is an effort to improve the phenoype and endotype characterisation of asthma to reduce the impact on patients’ quality of life, pulmonary function and negative treatment effects and to provide better control of the disease [5]. Thirty years after the onset of intercrisis treatment with inhaled medications, the rate of mortality and asthma control have improved. However, both national and international studies show that the number of uncontrolled patients remains high [6,7]. Several factors have been identified as being individually associated with asthma control [8] and many comorbidities, such as obesity, have been associated with negative effects on control [9]. In fact, to date, it is not understood why females appear to be more obese, severely affected and not atopic [10]. The aim of the present study was to evaluate the relationship between associated factors, asthma control and pulmonary function in adult women with asthma at the outpatient clinic of our hospital.

## Materials and Methods

### Study population

This cross-sectional study was conducted in an outpatient asthma clinic at University Hospital Gaffrée and Guinle (HUGG), Rio de Janeiro, with asthmatic women who were diagnosed with persistent asthma and assessed by GINA (Global Initiative for Asthma) [11] criteria between November 2013 and July 2014. The subjects were older than 18 years and were in routine follow up for more than one year. Asthma was diagnosed according to GINA criteria [11], using clinical data and lung function testing. Treatment had been initiated based on GINA guidelines [11], by the same physician with fixed doses at least three months prior to the study. All the patients had a BMI (Body Mass Index) between 18.5 and 39.9 kg/m^2^ (weight in kilograms divided by the square of height in meters). Subjects were excluded if they were current smokers or had smoked within past 5 years, were pregnant or nursing, had a history of psychiatric diseases, active pulmonary disease, malignancy, immunodeficiency, autoimmune diseases, cardiovascular disease, congestive heart failure, or thyroid dysfunction, were chronic users of systemic corticosteroids or immunosuppressive drugs or had airway infection or exacerbation during the four weeks preceding the study. Informed written consent was obtained from all subjects.

### Data collection

Demographic data [age and education (less or more than elementary school education)], duration of disease (less or more than 15 years), onset of disease (less or more than 12 years old), onset after menopause, asthma severity and control, anthropometric measurements (weight, height, waist circumference), use of drugs for gastroesophageal reflux disease treatment (GERD), and pulmonary function parameters such as pre-bronchodilator percent predicted FEV_1_ (forced expiratory volume in one second), FVC (forced vital capacity) and FEV_1_/FVC ratio were obtained.

### Assessment of asthma control

Asthma control was defined as fully controlled, partly controlled and not controlled according to GINA guidelines [11]. Patients answered two asthma control self-reported questionnaires: the Asthma Control Test (ACT) [12] and Asthma Control Questionnaire (ACQ) in the full version with seven questions [13] that was authorised for use. As cut-off points to identify asthma control, we used an ACQ score ≤ 0.75 and an ACT score ≥20.

### Assessment of severity of asthma

To identify disease severity, we used GINA 2002 classification, which divides patients into 4 categories: mild intermittent, mild, moderate and severe persistent, based on the frequency of symptoms, spirometry results and pharmacological treatment [14].

### Anthropometric measures

Weight and height were measured to the nearest 0.1 kg and 0.1 cm, respectively, according to standard protocols [15]. Waist circumference (WC) was measured to the nearest 0.1 cm, using a nonelastic tape measure, at the midpoint between the lower costal margin and iliac crest, at the end of normal expiration in supine position, with no clothes over the site of measurement, laterally extended arms and feet together [15]. All measurements were conducted by the same physician. BMI, waist circumference (WC), waist-to-height ratio (WHtR) and conicity index (CI) were calculated. BMI was categorised as normal, overweight and obese according to WHO (World Health Organization) criteria [15]. Central obesity was defined according to the measurements of WC, WHtR and CI. WC over 88 cm was defined as central obesity according to WHO criteria [15]. Central obesity was defined as a WHtR greater than 0.50 and CI greater than 1.18 and 1.22 for women 18–49 years and older than 50 years respectively [16].

### Assessment of respiratory function

Pulmonary function tests were performed by a single physician according to American Thoracic Society (ATS) guideliness [17] using the Spirometer Spiron 2 (Codax Corporation; São Paulo, Brazil), to determine the pre-bronchodilator predicted FEV_1_% and FVC% and FEV_1_/FVC ratio parameters. Recorded baseline FEV_1_ was the best among the three reproducible values in acceptable curves, with less than 5% amplitude. We used the predicted pulmonary function values of the Knudson standards [18]. The study was approved by the Research Ethics Committee of HUGG on 10 October 2013.

### Statistical analysis

A bivariate analysis was performed using the t-test for continuous variables and the chi-square test for categorical variables to investigate the relationship between clinical and demographic characteristics and asthma control. Variables showing an association with a p-value up to 0.20 in bivariate analysis were used in regression models. In logistic regression, the dichotomous dependent variable was asthma control (controlled and partially/not controlled). The results are expressed as odds ratios (OR) and 95% confidence intervals. When analysing the association of respiratory function parameters, the pre-bronchodilator predicted FEV_1_%, FVC%, and FEV_1_/FVC ratio were defined as dependent variables. The level of significance was set at 5%. All analyses were performed using SPSS statistical software, version 17.0.

## Results

The demographic and clinical characteristics of the subjects with total control versus those with partially controlled and uncontrolled asthma are shown in Table 1. One hundred twenty-four women were included, from 19 to 82 years old, with a mean age of 53.5 ± 14.0 years. A total of 57%, 38% and 21% of women were found to be totally controlled using, the ACT, the ACQ and GINA criteria, respectively. Based on BMI, 72% were overweight or obese. Central obesity was detected in 68% of subjects using WC and 84% using CI. There was a significant difference between asthma control and central obesity using WC, asthma severity and pulmonary parameters with both ACQ and GINA criteria. Age, education level, disease duration, onset of asthma, asthma after menopause, and treatment for GERD were not associated with asthma control. There was a significant difference in mean FEV_1_ and FEV_1_/FVC ratio according to asthma control with both the ACQ and GINA criteria, and in the FVC with all three control assessment tools.

**Table 1:** **Demographic and clinical characteristics of patients according to asthma control; HUGG, 2013-2014**

	**ACT**	**ACT**		**ACQ**	**ACQ**		**GINA**	**GINA**	
	**Controlled**	**Partially/uncontrolled**	**p value**	**Controlled**	**Partially uncontrolled**	**p value**	**Controlled**	**Partially/uncontrolled**	**p value**
Number(%)	71(57.26)	53(42.74)		47(37.90)	77(62.10)		26(20.97)	98(79.03)	
Age in years(SD)	53.56(14.61)	53.53(13.28)	0.85	51.40(16.31)	54.86(12.31)	0.21	48.31(16.27)	54.94(13.07)	0.06
BMI(SD)	27.69(4.64)	28.43(4.77)	0.39	27.55(4.49)	28.29(4.81)	0.39	26.77(5.02)	28.33(4.57)	0.16
Obesity(BMI)									0.06
Eutrophic(%)	20(28.1)	15(28.4)	0.17	15(32.0)	20(26.0)	0.70	12(46.16)	23(23.47)	
Overweight(%)	33(46.5)	17(32.0)		19(10.4)	31(40.2)		7(26.92)	43(43.88)	
Obese(%)	18(25.4)	21(39.6)		13(27.6)	26(33.8)		7(26.92)	32(32.65)	
Central Obesity(WC)									0.0086
Without obesity(%)	25(35.2)	14(26.4)	0.33	21(44.5)	18(23.4)	0.01	14(53.85)	25(25.51)	
With obesity(%)	46(64.8)	39(73.6)		26(55.5)	59(76.6)		12(46.15)	73(74.49)	
Central Obesity(WHtR)									0.36
Without obesity(%)	6(8.5)	2(3.8)	0.46	4(8.5)	4(5.0)	0.47	3(11.54)	5(5.11)	
With obesity(%)	65(91.5)	51(96.2)		43(91.5)	73(95.0)		23(88.46)	93(94.89)	
Central Obesity(CI)			0.09			0.00			0.0015
Without obesity(%)	15(21.1)	5(9.5)		14(29.8)	6(8.0)		10(38.46)	10(10.20)	
With obesity(%)	56(78.9)	48(90.5)		33(70.2)	71(92.0)		16(61.54)	88(89.80)	
Education Level			0.05			0.33			1.0
Less than 4 years(%)	31(43.6)	14(26.5)		20(42.5)	25(32.5)		9(34.62)	36(36.73)	
More than 4 years(%)	40(56.4)	39(73.5)		27(57.5)	52(67.5)		17(65.38)	62(63.27)	
Disease duration in years(SD)	26.08(17.13)	27.43(17.44)	0.66	25.13(16.68)	27(17.56)	0.43	21.61(15.44)	28(17.47)	0.07
Disease duration			0.69			0.64			0.06
Less than 15 years(%)	23(32.4)	15(28.3)		15(32.0)	23(29.9)		12(46.15)	26(26.53)	
More than 15 years(%)	48(67.6)	38(71.7)		32(68.0)	54(70.1)		14(53.85)	72(73.47)	
Age of onset			0.67			0.52			1.0
≤12years(%)	17(24.0)	15(28.3)		14(29.8)	18(23.4)		7(26.92)	25(25.51)	
>12 years(%)	54(76.0)	38(71.7)		33(70.2)	59(76.6)		19(73.08)	73(74.49)	
Asthma after menopause			0.65			0.64			0.59
Yes(%)	13(18.3)	12(22.6)		8(17.0)	17(22.0)		4(15.38)	21(21.43)	
No(%)	58(81.7)	41(77.4)		39(83.0)	60(78.0)		22(84.62)	77(78.57)	
Treatment of GERD			0.06			0.85			0.65
Yes(%)	23(32.4)	26(49.0)		18(38.3)	31(40.26)		9(34.62)	40(40.82)	
No(%)	48(67.6)	27(51.0)		29(61.7)	46(59.74)		17(65.38)	58(59.18)	
Asthma severity			0.50			0.04			0.0002
Mild(%)	10(14.0)	2(3.8)		8(17.0)	4(5.2)		8(30.77)	4(4.10)	
Moderate(%)	21(29.7)	11(20.7)		14(29.8)	18(23.4)		6(23.08)	26(26.52)	
Severe(%)	40(56.3)	40(75.5)		25(53.2)	55(71.4)		12(46.15)	68(69.38)	
FVC, % of predicted(SD)	90.22(18.38)	82.67(19.79)	0.03	94.89(18.67)	82.17(18.13)	0.0003	100.99(19.80)	83.28(17.44)	0.0002
FEV_1_ , % of predicted(SD)	72.92(20.42)	65.36(21.99)	0.05	80.91(19.57)	62.91(19.54)	<0.0001	88.23(18.83)	64.68(19.18)	<0.0001
FEV_1_/FVC ratio (SD)	79.93(12.62)	77.63(12.73)	0.32	84.70(10.98)	75.47(12.41)	0.0002	87.41(10.99)	76.65(12.15)	<0.0001

There was a significant difference between central obesity (WC) and asthma control using the ACQ and GINA criteria. We also found an association between central obesity (WC) and asthma control with the ACQ and GINA criteria after adjustment for the confounders (Table 2).

**Table 2:** **Logistic regression model showing odds ratios (OR) and confidence intervals (95% CI) for asthma control variables; HUGG, 2013–2014**

		**Asthma control**		
**Independent variable**	**Category or Increment**	Model 1* OR (95% CI)	Model 2** OR (95% CI)	Model 3*** OR (95% CI)
Age	Years	1.01 (0.98-1.05)	1.00 (0.98-1.00)	0.99 (0.96-1.02)
Education level	>4 years	Baseline	Baseline	Baseline
	≤4 years	0.51 (0.17-1.50)	0.46 (0.19-1.08)	0.45 (0.19-1.07)
Disease duration	Years	1.00 (0.97-1.04)	0.99 (0.97-1.02)	0.99 (0.97-1.01)
Asthma severity	Mild	Baseline	Baseline	Baseline
	Moderate/Severe	10.34 (2.56 -41.76)	3.53 (0.94-13.13)	4.52 (0.91-22.41)
Central obesity (WC)	Normal	Baseline	Baseline	Baseline
	>88 cm	3.15 (1.09-9.09)	2.53 (1.06-6.05)	1.70 (0.70-4.09)

*Model 1: assessment of asthma control using GINA criteria.

**Model 2: assessment of asthma control by the ACQ.

***Model 3: assessment of asthma control by the ACT.

In another regression model with the same variables but BMI instead of WC, there was only a strong correlation between asthma severity and the assessment of control using GINA criteria (Table 3).

**Table 3:** **Logistic regression model showing odds ratios (OR) and confidence intervals (95% CI) for asthma control variables; HUGG, 2013–2014**

		**Asthma Control**		
**Independent variable**	**Category or increment**	Model 1* OR (95% CI)	Model 2** OR (95% CI)	Model 3*** OR (95% CI)
Age	Years	1.02 (0.98-1.06)	1.10 (0.98-1.04)	1.00 (0.97-1.03)
Education level	>4 years	Baseline	Baseline	Baseline
	≤4 years	2.06 (0.69-6.12)	2.09 (0.89-4.87)	2.32 (0.97-5.53)
Disease duration	Years	1.01 (0.97-1.04)	1.00 (0.97-1.02)	0.99 (0.97-1.02)
Asthma severity	Mild	Baseline	Baseline	Baseline
	Moderate/Severe	8.93 (2.23-35.71)	3.31 (0.89-12.26)	4.34 (0.87-21.59)
BMI	Normal	Baseline	Baseline	Baseline
	Overweight/obese	2.73 (0.97-7.68)	1.05 (0.44-2.50)	1.10 (0.47-2.59)

* Model 1: assessment of asthma control using GINA criteria.

**Model 2: assessment of asthma control by the ACQ.

***Model 3: assessment of asthma control by the ACT.

There was also a significant association between asthma control, WC and severity using the GINA criteria but using ACQ there was only an association between asthma control and WC after adjustment for the other variables, including GERD in a stepwise logistic regression model (Table 4).

**Table 4:** **Stepwise logistic regression model showing odds ratios (OR) and confidence intervals (95% CI) for asthma control variables; HUGG, 2013–2014**

		**Asthma Control**	
**Independent Variable**	**Category**	Model 1* OR (95%CI)	Model 2** OR (95%CI)
Central Obesity (WC)	Normal	-	-
	>88 cm	3.55 (1.34-9.37)	2.55 (1.15-5.45)
Asthma	Mild	-	-
Severity	Moderate/Severe	10.34 (2.56-41.76)	

* Model 1: assessment of asthma control using GINA criteria.

** Model 2: assessment of asthma control by the ACQ.

In the linear regression models, there was a significant association between age and FEV_1_. In the model with FVC as the dependent variable, there was significant association between central obesity and age, and in the model with FEV_1_/FVC, there was only association with disease duration (Table 5).

**Table 5:** **Linear regression model of pre-bronchodilator predicted FEV**_**1**_ **%, FVC% and FEV**_**1**_ **/FVC ratio in asthmatic women; HUGG, 2013–2014**

		**FEV**_**1**_ **% predicted**		**FVC % predicted**		**FEV**_**1**_ **/FVC**	
**Variable**	**Category or increment**	Model 1* β (95% CI)	**p**	Model 2** β (95% CI)	**p**	Model 3*** β (95% CI)	**p**
Central	Normal	Baseline	0.20	Baseline	0.02	Baseline	0.57
Obesity	>88 cm	−5.67 (−14.48 to 3.13)		−8.98 (−16.75 to −1.20)		1.51 (−3.82to 6.85)	
Age	Years	−0.39 (−0.73 to 0.60)	0.02	−0.43 (−0,73 to −0,14)	0.00	−0.03 (−0.23 to 0.17)	0.75
Education Level	>4 years	Baseline	0.68	Baseline	0.46	Baseline	0.99
	≤4 years	−1.76 (−10.20 to 6.68)		−2.72 (−10.14 to 4.69)		−0.02 (−5.14 to 5.09)	
Age of Onset	≤12years	Baseline	0.82	Baseline	0.43	Baseline	0.36
	>12years	1.46 (−11.42 to 14.36)		4.46 (−6,.89 to 15.81)		−3.61 (−11.42 to 4.20)	
Disease Duration	Years	−0.07 (−0.40 to 0.25)	0.65	0.16 (−0.13 to 0.45)	0.27	−0,23 (−0.43 to −0.03)	0.02

*Model 1: Linear regression model pre-bronchodilator predicted FEV_1_%.

R^2^:0,075.

**Model 2: Linear regression model of pre-bronchodilator predicted FVC%.

R^2^:0,107.

***Model 3: Linear regression model of pre-bronchodilator FEV_1_/FVC ratio.

R^2^:0,032.

Central obesity defined according to WC.

## Discussion

The concept of asthma and its heterogeneity has recently changed due to the characterisation of different phenotypes that are associated with different expression profiles, disease severity and disease control [5,19]. Many phenotypes associated with asthma have been described that are defined by multivariate features, including clinical, genetic and molecular features; the phenotypes are able to interact, have different behaviours regarding control and response to treatment and are linked to gender [5,10,19]. During childhood, asthma is more prevalent in males, after puberty, however, its incidence increases in women [20]. In addition to being more affected by asthma, women are at greater risk of developing non-atopic asthma, which has been associated with greater severity and poor control [21,22]. However, the determinants for this greater risk among women are not yet known. Therefore, we decided to study only females, who, different from men, are influenced by hormonal changes during their entire lives, resulting in worsening of symptoms, decreased lung function, and dysregulation of the immune response [22]. We believe that this is the first study that associates asthma control and its comorbidities, including central obesity, only in women, using 3 different control assessment tools.

Despite many advances in asthma management, the majority of both national and international studies show that at least 40% of patients are not fully controlled [6,7]. In our study, we also observed that regardless of the control assessment tool used, more than 40% of participants were partly and not controlled; this was most notably observed with the GINA assessment, followed by ACQ. We believe that the reason for this high frequency is because the patients in our study were from a reference health centre, which most likely has a greater number of patients with severe disease.

We believe that the full version of the ACQ was most similar tool to the GINA criteria results because both offer a more objective and comprehensive assessment of disease control, using a respiratory functional component, the FEV_1_. There are at least 17 different validated instruments with rating scales to assess asthma control, and the ACT and ACQ are the most frequently used and validated [23]. The choice of the control assessment tool depends on the objectives, personal preference, and the support structure. The GINA classification is perhaps the most widely recognised and recommended asthma assessment tool worldwide [11]. Although the GINA classification has not been formally validated, recent studies have demonstrated different degrees of agreement between it and other tools [24-26]. When Rodrigo et al. [25] tested the Spanish version of the ACT, they observed that the instrument had a weak correlation with FEV_1_ and that almost 40% of patients with a predicted FEV_1_ less than 60% had considered his illness totally controlled, concluding that the ACT is not an appropriate tool to guide asthma management if used without spirometry. In addition, a recent systematic review that included 21 trials and 11,141 patients showed that compared to GINA classification, the ACT and ACQ were not useful in identifying uncontrolled asthma [26]. Therefore, we should not be surprised that control instruments based solely on subjective assessment (for example, the ACT) have little agreement with those that use objective measures.

We also observed that asthma severity was associated with control in the bivariate model only when the assessment was conducted with the ACQ and the GINA classification. In this evaluation, the treatment could control the disease but not interfere with its severity [14]. Although severity and asthma control are used interchangeably in some studies and have overlapping characteristics, they are quite different concepts. Severity reflects the intensity of treatment required to achieve disease control. In contrast, asthma control incorporates clinical control, risk of exacerbations and decline in lung function [11,14,27]. We believe that our results were influenced by the age of patients, with a consequent reduction in lung function, in addition to the greater severity of their disease.

To date, it is not known if there is a pathophysiological association between asthma and obesity or if this association is overestimated due to changes in respiratory function such as reduced lung volumes [28], thoracic limitation [29], increased oxygen respiratory demand [30], and increased comorbidities risk (GERD,OSA), regardless of the presence of asthma [9,31]. Aaron et al’s longitudinal study [32] in Canada showed that nearly one-third of obese individuals assessed by BMI and one-third of non-obese patients with an initial diagnosis of asthma were not considered asthmatics when reassessed clinically and functionally with spirometry and a bronchial challenge test. When we excluded patients with level III obesity, we aimed to reduce the interference of severe obesity in the interpretation of questionnaires, reducing the impact on the overestimation of the symptoms of asthma, and respiratory function tests. To date the mechanisms of association between asthma and obesity, GERD and OSA are not known [9,31,33]. Our findings indicate that asthma control and obesity are associated, independent of GERD; this is similar to the results of a study by Dixon et al. who found that GERD was not associated with worse asthma control in obese patients [34]. Few studies to date, mostly crossover studies, have investigated the association between central obesity and asthma [35,36]. Our results showed that central obesity and not excess body fat was associated with asthma control in women. BMI cannot distinguish between fat mass and muscle mass [37]. Moreover, BMI does not predict abdominal fat deposition, which is associated with reduced pulmonary function, metabolic syndrome, cardiovascular complications and, possibly, poor asthma control [37,38]. A group of asthmatic women who were partially/uncontrolled according to the GINA assessment had central obesity and higher disease severity, even considering age, education, onset and duration of disease. These results are similar to those of other studies that have clustered clinical, inflammatory and functional profiles pointing to a more symptomatic asthma associated with obesity in women [5,19]. However, to date, inflammatory markers have not been associated with phenotypes, and the studies have differing results on the levels of eosinophilic inflammation [5,19]. The differences in pathophysiological mechanisms, levels of inflammation and immune dysregulation might possibly result from hormonal changes and a high concentration of leptin, considered a potent inflammatory adipokine, that is associated in particular with subcutaneous and central fat distribution and is found in higher levels in women and obese individuals [39].

Furthermore, we conclude that in addition to the inverse association with age, there is a negative association between FVC and central obesity, independent of the age of onset and disease duration. Although the association between central obesity and lung function has been studied in the general population, few studies have been conducted in the asthmatics population [33,40]. In addition to being determined by age, gender, and height [18], lung volumes seem to be more negatively affected by central obesity than overall obesity and peripheral fat [41]. Our results are similar to a recently published meta-analysis that showed a more significant negative association between central obesity and FVC compared to FEV_1,_ both in women and men, with no change in the FEV_1_/FVC ratio [41]. However, the authors concluded that airway obstruction cannot be entirely ruled out, even when associated with restrictive pulmonary patterns [41]. To date, it is not known whether this association is mechanical, related to an asthma inflammatory mechanism due to fat accumulation in the abdomen limiting diaphragm movement [28,29] or inflammatory related to the action of leptin and other cytokines such as IL-6 and TNF-alpha produced by visceral fat [39], that are possibly capable of increasing airway hyperresponsiveness in obese asthmatics [42]. This study has some limitations, such as the possibility of having included more severely affected and less well-controlled patients, because our hospital is a referral centre for the treatment of asthma. In addition, it was not possible to explain any causal relationship because of the nature of the study.

## Conclusions

In conclusion, our study demonstrates that women with poorly controlled asthma have more severe disease and increased obesity. In addition, the study demonstrates that there is a negative relationship between central obesity and asthma control assessed using GINA criteria and the ACQ controlling for other independent variables. Furthermore, compared to the GINA classification, the ACT seems not to be a good instrument to assess women with asthma that are not well controlled. The study also emphasises the importance and complexity of defining asthma control precisely, and we suggest further prospective studies to control for a possible intervention of comorbidities that can emerge during the course of the disease. We also believe that women should be studied as a separate group, because they are influenced by continuous hormonal variations and are affected by a more severe inflammatory asthma. Finally, we recommend studies that investigate inflammatory markers, such as cytokines, and fat distribution to define phenotypes that could be associated with central obesity and asthma control.

## Abbreviations

ACT: Asthma control test
ACQ: Asthma control questionnaire
ATS: American thoracic society
BMI: Body Mass Index
CI: Conicity index
DATASUL: National Health System data
FEV_1_%: Forced expiratory volume percentage in one second
FVC%: Forced vital capacity percentage
GINA: The global initiative for asthma
GERD: Gastroesophageal reflux disease
HUGG: University Hospital Gaffrée and Guinle
WC: Waist circumference
WHO: World Health Organization
WHtR: Waist-to-height ratio
